# Brief Treatment of Co-Occurring Post-Traumatic Stress and Depressive Symptoms by Use of Accelerated Resolution Therapy^®^

**DOI:** 10.3389/fpsyt.2013.00011

**Published:** 2013-03-08

**Authors:** Kevin E. Kip, Kelly L. Sullivan, Cecile A. Lengacher, Laney Rosenzweig, Diego F. Hernandez, Rajendra Kadel, Frank A. Kozel, Amy Shuman, Sue Ann Girling, Marian J. Hardwick, David M. Diamond

**Affiliations:** ^1^College of Nursing, University of South FloridaTampa, FL, USA; ^2^Balanced Living PsychologyTampa, FL, USA; ^3^Department of Psychiatry and Neurosciences, University of South FloridaTampa, FL, USA; ^4^Western New England UniversitySpringfield, MA, USA; ^5^Research and Development Service, Veterans Affairs HospitalTampa, FL, USA; ^6^Department of Psychology, Center for Preclinical and Clinical Research on PTSD, University of South FloridaTampa, FL, USA; ^7^Department of Molecular Pharmacology and Physiology, Center for Preclinical and Clinical Research on PTSD, University of South FloridaTampa, FL, USA

**Keywords:** psychological trauma, PTSD, depression, exposure therapy, eye movements, brief treatment

## Abstract

This uncontrolled prospective cohort study evaluated the use of accelerated resolution therapy (ART) for treatment of comorbid symptoms of post-traumatic stress disorder (PTSD) and major depressive disorder. Twenty-eight adult subjects, mean age of 41 years (79% female, 36% Hispanic), received a mean of 3.7 ± 1.1 ART treatment sessions (range 1–5). ART is a new exposure-based psychotherapy that makes use of eye movements. Subjects completed a range of self-report psychological measures before and after treatment with ART including the 17-item PCL-C checklist (symptoms of PTSD) and 20-item Center for Epidemiologic Studies Depression Scale (CES-D). For the PCL-C, the pre-ART mean (±standard deviation) was 62.5 (8.8) with mean reductions of −29.6 (12.5), −30.1 (13.1), and −31.4 (14.04) at post-ART, 2-month, and 4-month follow-up, respectively (*p* < 0.0001 for comparisons to pre-ART score). Compared to pre-ART status, this corresponded to standardized effect sizes of 2.37, 2.30, and 3.01, respectively. For the CES-D, the pre-ART mean was 35.1 (8.8) with mean reductions of −20.6 (11.0), −18.1 (11.5), and −15.6 (14.4) at post-ART, 2-month, and 4-month follow-up, respectively (*p* ≤ 0.0001 compared to Pre-ART score). This corresponded to standardized effect sizes of 1.88, 1.58, and 1.09, respectively. Strong correlations were observed at 2-month and 4-month follow-up for post-treatment changes in PTSD and depression symptom scores (*r* = 0.79, *r* = 0.76, respectively, *p* ≤ 0.0002). No serious treatment-related adverse effects were reported. In summary, ART appears to be a promising brief, safe, and effective treatment for adults with clinically significant comorbid symptoms of PTSD and depression. Future controlled and mechanistic studies with this emerging therapy are warranted, particularly given its short treatment duration, and in light of current heightened emphasis on health care cost constraints.

## Introduction

Post-traumatic stress disorder (PTSD) is a prevalent, disabling anxiety disorder characterized by re-experiencing, avoidance, numbing, or arousal that may occur after witnessing or experiencing a traumatic event (American Psychiatric Association, 2000). The lifetime prevalence of PTSD among adults in the U.S. has been estimated at 6.8% (Kessler et al., 2005). PTSD is often compounded by comorbid diagnoses of other mood disorders, particularly depression. Data from the National Comorbidity Survey estimate a correlation between lifetime major depressive disorder (MDD) and lifetime PTSD of 0.50 (Kessler et al., 1995). The prevalence of comorbid PTSD and depression has been studied in populations who have experienced a range of traumas. Among 426 participants in the Veterans’ Health Study who met criteria for PTSD, 82% also met criteria for depression (Hankin et al., 1999). A study of 62 survivors of motor vehicle accidents with PTSD reported comorbid depression in 53% of participants (Blanchard et al., 1998). Similarly, studies of patients hospitalized for physical trauma have found comorbid depression in 43 (Shalev et al., 1998) to 55% (O’Donnell et al., 2004) when PTSD was present 3–4 months following the traumatic experience.

The strong association between PTSD and depression suggests a common vulnerability following a traumatic experience (Breslau et al., 2000). It has been postulated that symptoms of depression and PTSD may actually represent a singular construct (O’Donnell et al., 2004), a premise supported by Rasch modeling (Elhai et al., 2011). Furthermore, the presence of DSM criterion A for PTSD diagnosis (i.e., presence of a traumatic event) has been recently shown to be the only distinguishing symptom between patients with MDD versus PTSD (Gros et al., 2012). Still, the overlap between symptoms of PTSD and MDD is not absolute, and disparate views on the singular construct exist (Grant et al., 2008). Variation in extent of symptom overlap has been shown in veterans with PTSD where the specific PTSD symptoms of numbing and dysphoria are more closely related to symptoms of MDD than other PTSD symptoms of intrusions, avoidance, or arousal (Gros et al., 2010). Importantly, in terms of etiology and potential treatment approaches, examination of data from The National Comorbidity Survey has suggested that PTSD is more often the primary disorder temporally among people with both PTSD and depression (Kessler et al., 1995).

Given the high prevalence of comorbid PTSD and depression, as well as the overlap in symptomatology, the impact of comorbid diagnoses should be considered and addressed in treatment. The co-occurrence of PTSD and MDD is associated with greater disorder severity (Kessler et al., 2005). In a study of PTSD symptoms among veterans at a primary care clinic, those with either MDD or PTSD (as a singular diagnosis) showed similar levels of PTSD symptoms on the PCL-C rating scale. Veterans with both PTSD and MDD had more severe symptoms on all PCL-C subscales (intrusions, avoidance, dysphoria, numbing, and hyperarousal) compared to patients with either disorder alone (Gros et al., 2012). In addition to its association with greater PTSD symptom severity, comorbid PTSD and depression has been associated with greater anxiety and lower global functioning (Blanchard et al., 1998; Momartin et al., 2004; Nixon et al., 2004).

Given greater disorder severity, patients with comorbid diagnoses have higher health service utilization compared to those with individual conditions (Kramer et al., 2003), and they also respond differently to depression treatment regimens. In a study of low income women, depression was treated with cognitive behavioral therapies (CBT), anti-depressant medications, or community mental health referral. Through 10 months of treatment, symptoms of depression improved less in the women with comorbid PTSD than in those with singular depression (Green et al., 2006). Cognitive processing therapy (CPT), which entails 12 sessions (60–90 min) with practice of skills outside of sessions, has been reported to be effective in treating comorbid PTSD and depression among female assault victims (Nishith et al., 2005). Still, nearly all of the literature on treatment for PTSD and comorbid depression has arisen in the past few years (Foa et al., 2009), and integrated treatment is generally recommended.

Notwithstanding the general recommendation for integrated psychotherapy and pharmacotherapy, results to date suggest that for individuals with comorbid PTSD and depression, psychotherapy may be most effective if specifically targeted to PTSD rather than depression as the primary disorder (e.g., Kessler et al., 1995). In this realm, evidence-based CBT that are formally endorsed as first line treatment for PTSD, including by the Veterans Administration and Department of Defense, include CPT, exposure-based therapy (e.g., Prolonged exposure (PE) therapy), and eye movement desensitization and reprocessing (EMDR) (U. S. Department of Veterans Affairs, 2010). Nonetheless, the above mentioned therapies have notable limitations including variable efficacy, frequent dropout, and lengthy treatment regimens. In brief, PE consists of 10 sessions (approximately 90 min each) with corresponding homework assignments (Foa et al., 2007), dropout rates of approximately 0–50% (Hembree et al., 2003; Schnurr et al., 2007; Schottenbauer et al., 2008), and non-response rates between 20 and 67% (Minnen and Hagenaars, 2002; Schottenbauer et al., 2008). CPT is delivered over 12 sessions lasting 60–90 min with practice of skills outside of therapy sessions (Resick and Schnicke, 1996), dropout rates of approximately 4–29% (Hembree et al., 2003; Schottenbauer et al., 2008), and non-response rates between 4 and 48% (Schottenbauer et al., 2008). EMDR consists of 8–12 weekly 90-min sessions (Friedman, 2003), dropout rates of approximately 0–36%, and non-response rates between7 and 92% (Schottenbauer et al., 2008). Hence, no single approach has been shown to be optimal in terms of delivery time, treatment completion, and amelioration of PTSD symptoms. In addition, exacerbation rates (i.e., of symptoms) have been reported to range between 13 and 28% for PE and 5 and 10% for CBT (Schottenbauer et al., 2008).

In light of the above treatment limitations, in particular, sheer length (and hence cost) of treatment, we report on a new, brief exposure-based psychotherapy known as Accelerated Resolution Therapy (ART^®^) for treatment of PTSD among adults with comorbid symptoms of depression. As described below, this therapy has a strong theoretical basis coupled with limited empirical base for symptoms of PTSD at large (i.e., irrespective of depressive symptoms) (Kip et al., 2012). Through the delivery of ART for trauma-related experiences and distress, we hypothesized clinically significant reductions in symptoms of both PTSD and depression in a brief treatment period. We further hypothesized close concordance in treatment response with respect to both PTSD and depression.

### Description of ART protocol

The ART protocol uses cognitive behavioral and experiential therapies and was developed to treat both physiological and cognitive aspects of PTSD, which as a disorder, has been described as a consequence of failed memory processing when the brain fails to appropriately consolidate and integrate *episodic* memory into the *semantic* memory system (Stickgold, 2002). The two major components of ART that draw from existing evidence-based therapies for PTSD include the practices of *imaginal exposure* (reliving) and *imagery rescripting*. This is based on the tenets that: (i) most intrusive memories (i.e., as in PTSD) involve sensory imagery with visual material being most common (Hackmann, 2011); (ii) PTSD memories are not well integrated (Conway and Pleydell-Pierce, 2000) and to become less intrusive, need to be integrated with more positive images (Conway et al., 2004); and (iii) changes in imagery tend to be accompanied by larger affect shifts than changes in verbal thought (Holmes et al., 2006).

With respect to the imaginal exposure component prompted by the ART clinician and retrieved by the patient (whether verbalized or not), which in and of itself can bring about cognitive change in the setting of PTSD (Foa et al., 1995), the ART clinician oscillates their hand at eye level to the patient (sets of 40 at a time). In conjunction with these oscillations, the patient performs lateral left-right eye movements to facilitate the separation (elimination) of physiological sensations associated with purposeful recall of traumatic experiences prior to cognitive intervention. In ART, this is referred to as “processing” of sensations, and it is believed to have a calming effect that may promote subsequent limbic change by shifting from a dominant sympathetic nervous system mode during imaginal exposure (i.e., “flight or fight” response) to complementary parasympathetic nervous system dominance.

After exposure to imagined material and processing of physiological sensations for a given traumatic scene, the ART clinician facilitates imaging rescripting (IR) by asking the patient to come up with a “solution” to “replace” the distressing images and/or other senses (but not narrative memory) with positive images. In ART, this technique is known as Voluntary Image Replacement (VIR) and it directly parallels IR (“Type A”) in which a preexisting negative mental image is transformed into a more benign image (i.e., negative image to positive image through rescripting) (Holmes et al., 2007), and which has been successfully used to treat survivors of traumatic industrial accidents suffering from PTSD (Grunert et al., 2007).

Despite similarities with EMDR, the most poignant differences between ART and EMDR are: (i) *images*: ART uses the VIR technique to change the actual recall of images and/or other senses (i.e., from negative to positive), whereas EMDR aims to cognitively desensitize the patient about their trauma (images); (ii) *sensation processing*: ART spends considerably more time processing physiological sensations invoked by exposure to imagined material than EMDR, and by protocol, dictates that after each “scene-focused” set of eye movements, the therapist use a corresponding set of eye movements specifically to process (neutralize) physiological sensations; (iii) *standardization*: for each set of eye movements, ART uses a fixed number (40) to help the patient process, but not be flooded with information, whereas EMDR changes the number of eye movements. In addition, ART clinicians use a set of standard interventions and a fidelity checklist from the ART training manual, whereas EMDR is less standardized and may require the therapist to come up with their own “cognitive interweave” when they get “stuck.” Thus, unlike EMDR, ART is purposely not free-associative. In addition, the use of the VIR technique in ART aims to change the images and other sensations associated with the problematic memory; this differs from PE therapy which aims to decrease the distress associated with trauma-related thoughts and prior exposure.

The length of treatment with ART is brief yet variable, and is based on processing (treating) one or more traumatic scenes identified as contributing to symptoms of PTSD. This is consistent with the protocol of Arntz and Weertman (1999) who stress that there is no need for PE, *per se*, in the treatment of PTSD. When multiple traumatic scenes exist, approximately three representative scenes (i.e., those most contributing to symptoms) may be processed to eliminate presenting sensations and symptoms, with an apparent generalizing effect to any remaining scenes of lesser clinical importance. Thus, treatment is completed when the scene or representative scenes are processed and the patient reports significant or full relief from symptoms associated with the problematic memory. In this study, consenting participants underwent between one and five sessions of ART (within 3 weeks), each approximately 60–75 min in length. Individual components used within each ART session have been described (Kip et al., 2012). All therapists completed a 42-item ART fidelity checklist after each treatment session, although, there was no recording of treatment sessions or independent verification of protocol-compliant delivery of ART.

## Materials and Methods

### Ethics statement

The study protocol was approved by the Institutional Review Board at University of South Florida (USF) and all participants provided written informed consent.

### Screening and enrollment

The present study is a subgroup analysis derived from a federally funded prospective cohort treatment study of adults with symptoms of PTSD initiated at the USF in 2011 (Kip et al., 2012). Participants, which included both veterans and civilians, were recruited through caseload referrals from licensed mental health therapists in the study area, flyers and brochures, direct meetings with providers of mental health services, and as a result of local media coverage. All participants were treated at the USF College of Nursing and received $20 for completion of self-report questionnaires (see below) before and after treatment and at 2- and 4-month follow-up.

For screening and potential enrollment, participants completed three instruments by written self-report as part of an initial intake assessment. This included the 17-item PCL-C Checklist (Weathers et al., 1993; Blanchard et al., 1996), a self-developed 9-item ART Intake Questionnaire, and the 125-item (yes/no) Psychiatric Diagnostic Screening Questionnaire (PDSQ). The PDSQ was used to screen for Axis I disorders and serve as a baseline assessment of psychopathology. This instrument has been validated against diagnostic criteria and interview-derived diagnoses over the course of 10 years and more than 3,000 administrations (Zimmerman and Mattia, 2001; Zimmerman and Chelminski, 2006). It can be quickly hand scored to obtain a total score (which functions as a global indicator of psychopathology) plus subscale scores for 13 disorders: MDD, generalized anxiety disorder (GAD), panic disorder, PTSD, alcohol abuse/dependence, drug abuse/dependence, psychosis, bulimia/binge-eating disorder, somatization disorder, obsessive-compulsive disorder, social phobia, hypochondriasis, and agoraphobia. Participants were given instructions by the study coordinator and completed these self-report forms in private (no proxy reports were allowed). Completion of these instruments was followed by a clinical interview between the potential study subject and an ART assessment clinician to determine study eligibility. “Screen fail” participants (i.e., those not meeting enrollment criteria, including those with a *T*-score >70 indicating need for a higher level of care) were offered a direct referral for counseling in the community, or two complimentary sessions at the study site (and appropriate referral thereafter) of an empirically based method of psychotherapy.

Full inclusion criteria for the parent study which aimed to treat symptoms of PTSD (i.e., irrespective of depression), and on which this subset analysis is based, were: (i) between the age of 21–60 years inclusive; (ii) symptoms indicative of PTSD, as defined as a score of >40 on the PTSD Checklist (range 17–85) – Civilian version (PCL-C) (Weathers et al., 1993; Blanchard et al., 1996), or in the absence of a score >40, other documented evidence of symptoms, including a high PTSD subscale score and/or endorsement of specific PTSD item responses on the PDSQ; (iii) ability to read and speak English to complete survey questions; and (iv) denial of suicidal and homicidal ideation or intent and no evidence of psychotic behavior or otherwise being in psychological crisis. Exclusion criteria for the parent study were: (i) brain injury prohibiting speech, writing, and purposeful actions; (ii) current suicidal ideation; (iii) major psychiatric disorder (e.g., bipolar disorder) primary to symptoms of psychological trauma; (iv) current treatment for substance abuse; (v) previous diagnosis of eye movement disorder anticipated to interfere with treatment (e.g., amblyopia); and (vi) any medical condition that, in the judgment of the principal investigator and/or ART therapist, may place the individual at high risk due to a potential heightened emotional reaction (e.g., previous heart attack, seizure disorder).

For the present subgroup analysis, additional inclusion criteria (beyond the parent study, Kip et al., 2012) were selected for analytical purposes to document evidence of both clinically significant PTSD and depression, and to approximate clinical diagnoses. This was based on scores on the PCL-C, Center for Epidemiologic Studies Depression Scale (CES-D), and subscale scores and individual item responses from the PDSQ. Specifically, eligibility for the subgroup analysis from the parent study required presence of all of the following: (i) PTSD: score of ≥44 on the PCL-C (Blanchard et al., 1996); score of ≥5 on the PTSD subscale of the PDSQ (Zimmerman and Mattia, 2001); and PDSQ endorsement of item #29 (ever experienced traumatic event) and/or item #30 (ever witnessed traumatic event); (ii) depression: score of ≥9 on the MDD subscale of the PDSQ (Zimmerman and Mattia, 2001); and score of ≥16 on the CES-D (McDowell and Newell, 1996).

### Therapist training

All therapists underwent intensive training in ART conducted in person by the developer (Laney Rosenzweig) and lead trainer (Amy Shuman) and in accordance with the ART training manual. This included two 8-h days on the theory, principles, and protocol for conducting ART including intake assessment, intervention techniques, eye movement regimen, challenges and solutions, and closure techniques. This was followed by directly observed supervised practice, and then follow-up training and assessment.

### Data collection

After written consent and clinical screening for determination of study eligibility, participants completed a demographic and brief medical history questionnaire provided to them by the research coordinator. In addition, baseline completion of self-reported outcome measures by study subjects (in addition to the previously completed PCL-C) included the following measures: 18-item Brief Symptom Inventory (BSI) (Derogatis, 2001); 20-item CES-D (Radloff, 1977); 21-item State-Trait Inventory for Cognitive and Somatic Anxiety (STICSA) (Ree et al., 2008); 26-item Self-Compassion Scale (SCS) (Neff, 2003); 29-item Aggression Questionnaire (AQ) (Buss and Perry, 1992); 10-item Alcohol Use Disorder Identification Test (AUDIT) (Saunders et al., 1993); 32-item Trauma-Related Guilt Inventory (TRGI) (Kubany, 1996); 21-item Post-Traumatic Growth Inventory (PTGI) (Tedeschi and Calhoun, 1996); and the Pittsburgh Sleep Quality Index (PSQI) (Buysse et al., 1989). These measures were the same as those included in the parent study and were selected to assess a wide range of psychological treatment response and on the basis of established reliability and validity. The outcome measures were completed by written self-report by study subjects immediately before the first ART session, after the final ART session, and at 2- and 4-months post-treatment. Post-treatment evaluations were completed in person except in rare instances when the subject could not come to the study site; in these instances, participants completed and returned assessments via U.S. mail. Occurrence of adverse events was inquired by the treating therapist prior to each ART session including the nature and intensity of each event, subsequent treatment actions, and judgment as to whether the event was causally related to use of ART.

For this subgroup analysis, changes in the PCL-C and CES-D were considered the primary outcomes, with all other measures treated as secondary outcomes. The PCL-C is a 17-item self-report measure of DSM-IV symptoms of PTSD (Weathers et al., 1993; Blanchard et al., 1996). Respondents rate how much they were “bothered by that problem in the past month.” Items are rated on a 5-point scale ranging from 1 (“not at all”) to 5 (“extremely”). A total score (range 17–85) can be obtained by summing the scores from each of the 17 items. The CES-D is a 20-item self-report scale which measures the current level of depressive symptomatology in the general population, with an emphasis on depressed mood during the past week (Radloff, 1977). Items are rated on a 4-point scale ranging from 0 (rarely or none of the time) to 3 (most or all of the time) with a possible range of 0–60. The CES-D incorporates the main symptoms of depression and was derived from five validated depression scales including the Beck Depression Inventory.

### Statistical methods

Continuous variables are expressed as mean ± standard deviation (SD); categorical variables are presented as percentages. The analysis is presented sequentially, initially, among the 44 individuals with evidence of clinically significant PTSD and depression, and second, among the 28 individuals who were eligible for the study (i.e., based on all inclusion criteria), enrolled, treated, and with follow-up data. For these 28 subjects, demographic and presenting characteristics were compared by pre-treatment PCL-C score (classified as below or above the median of 63) and pre-treatment CES-D score (classified as below or above the median of 36) using student *t* tests for continuous variables and Fisher’s Exact test for categorical variables. Standardized effect sizes for outcome measures were calculated as: (mean before ART – mean after ART)/SD of treatment difference scores (Morris and DeShon, 2002) with corresponding 95% confidence intervals calculated. Within-subject changes on scores from the PCL-C and CES-D were formally tested by repeated measures analysis of variance specifying an exchangeable correlation structure. Finally, the strength of association between corresponding pre- to post-ART changes in PTSD (PCL-C) and depression (CES-D) was estimated by use of Pearson correlation coefficients. This included changes in total score on the PCL-C, as well as subscale scores derived from a published factor analysis (Asmundsona et al., 2000) with four factors (subscales) as follows: intrusion/re-experiencing (items 1–5); avoidance (items 6–7); numbing (items 8–12); and hyperarousal (items 13–17). Due to multiple comparisons, a two-tailed *p*-value of <0.025 was used to define statistical significance for the primary outcomes (PCL-C and CES-D) and <0.001 for all secondary outcomes.

## Results

**Figure 1:**
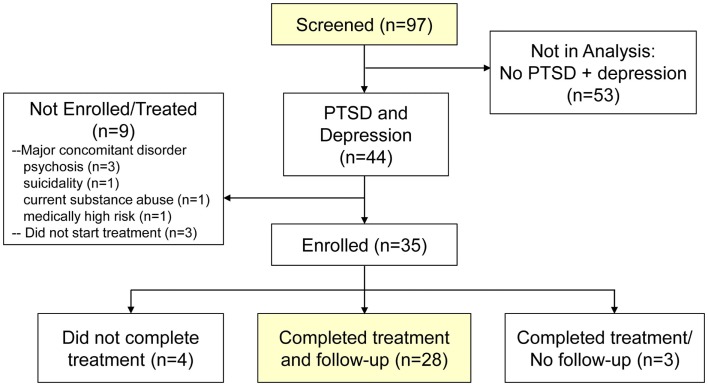
**Flow diagram of screening, enrollment, and treatment completion of study participants**.

From the parent study, a total of 97 individuals were screened, of whom, 44 (45.4%) met the sub-study definition for comorbid PTSD and depression (Figure 1). The 44 individuals with comorbid PTSD and depression were of similar mean age to the other 53 screened individuals (40.6 versus 41.1 years) and gender (76.3 versus 78.6%), yet predictably presented with a higher mean PDSQ-T score (60.2 versus 50.4, *p* < 0.0001). Of the 44 individuals with comorbid PTSD and depression, 9 (20.5%) did not meet eligibility (enrollment) criteria in the parent study (e.g., due to major concomitant disorder, such a psychosis or suicidality) or start treatment with ART. Of the 35 subjects enrolled, 4 (11.4%) did not complete treatment, 3 (8.6%) completed treatment but were lost to follow-up, and the remaining 28 (80.0%) completed treatment and had 2-month and/or 4-month follow-up data.

### Presenting psychological status

For the 9 of 44 individuals with clinical evidence of PTSD and depression, yet who were ineligible or did not start treatment with ART, concomitant psychopathology was highly prevalent, as determined from individual subscale scores on the PDSQ (Table 1). This included scoring at or above the cutpoint for bulimia/binge-eating disorder (44.4%), obsessive-compulsive disorder (OCD) (44.4%), panic disorder (66.7%), agoraphobia (75.0%), social phobia (88.9%), GAD (77.8%), somatization disorder (77.8%), psychosis (44.4%), and suicidal ideation in the past 2 weeks (55.6%). For subjects enrolled in the present study (*n* = 35), the extent of comorbid psychopathology was lower yet still prevalent for OCD, panic disorder, agoraphobia, social phobia, GAD, and somatization disorder.

**Table 1:** **Psychological status of screened consenting individuals with evidence of clinically significant PTSD and depression**.

Psychological measure	Ineligible/not treated^a^ (*n* = 9)	Enrolled; no follow-up^b^ (*n* = 7)	Treated; followed^c^ (*n* = 28)
Subscale of PDSQ (% meeting cutoff score)^d^			
Bulimia/binge-eating disorder (10/7)	44.4	14.3	25.0
Obsessive-compulsive disorder (7/1)	44.4	71.4	64.3
Panic disorder (8/4)	66.7	28.6	57.1
Psychosis (6/1)	44.4	42.9	42.9
Psychosis (6/2)^e^	11.1	14.3	10.7
Agoraphobia (11/4)	75.0	42.9	59.3
Social phobia (15/4)	88.9	71.4	89.3
Alcohol abuse/dependence (6/1)	11.1	0.0	25.0
Drug abuse/dependence (6/1)	22.2	0.0	25.0
Generalized anxiety disorder (10/7)	77.8	85.7	71.4
Somatization disorder (5/2)	77.8	57.1	57.1
Hypochondriasis (5/1)	33.3	71.4	46.4
Suicidal ideation in past 2 weeks^f^	55.6	42.9	28.6
PDSQ-T score (mean ± SD)	64.0 ± 6.8	57.0 ± 6.6	59.8 ± 7.3
PCL-C score (mean ± SD)	70.2 ± 6.3	66.4 ± 7.1	62.5 ± 8.8
CES-D score (mean ± SD)	33.6 ± 8.7	40.3 ± 7.0	35.1 ± 8.8

*^a^Reasons included psychosis (*n* = 3), suicidality (*n* = 1), current substance abuse (*n* = 1), medically high risk (*n* = 1), and did not start treatment (*n* = 3). ^b^Four participants started yet did not complete treatment and three participants completed treatment but did not have follow-up. ^c^Completed treatment and had 2- and/or 4-month follow-up assessment. ^d^Parentheses include number of subscale items/number required to meet cutoff score. ^e^Modified to require endorsement of two items on the PDSQ. ^f^Items 20 and 21 on the PDSQ: during the past 2 weeks; did you seriously consider taking your life? (Item #20); did you think about a specific way to take your life? (Item #21)*.

For the 7 of 35 subjects who did not complete treatment or have follow-up data, no major adverse events were reported. Compared to the 28 subjects treated and followed, the 7 subjects without full treatment and follow-up data were of similar age (33.6 ± 8.8 versus 40.5 ± 9.2, *p* = 0.08), female gender (71.4 versus 78.6%, *p* = 0.65), Hispanic ethnicity (33.3 versus 35.7%, *p* = 1.0), having had traumatic memories for ≥11 years (71.4 versus 82.1%, *p* = 0.61) and prior treatment for PTSD (71.4 versus 75.0%, *p* = 1.0). However, the 7 non-completers were less likely to be married than the 28 completers (14.3 versus 60.7%, *p* = 0.04). For the 28 subjects treated and followed, the mean PDSQ-T score was 59.8 ± 7.3 with mean scores on the PCL-C (62.5 ± 8.8) and CES-D (35.1 ± 8.8) approximately two SDs above established cutpoints that are suggestive of a diagnosis of PTSD (Blanchard et al., 1996) and depression (McDowell and Newell, 1996), respectively. For these subjects, the mean number of ART sessions was 3.7 ± 1.1 (range 1–5).

### Demographic and presenting characteristics

The mean age of the 28 treated and followed subjects was 40.5 ± 9.2 with 79% of female gender, 93% of Caucasian race, and 36% of Hispanic ethnicity (Table 2). Eighty-two percent had existing traumatic memories for ≥11 years and three-quarters had received prior mental health treatment for PTSD. When evaluating presenting characteristics by PCL-C scores below (*n* = 15) versus above (*n* = 13) the median (scores of 45–63 versus 64–81), the prevalence of higher PCL-scores was non-significantly related to male gender, having a history of hypertension, and having a history of neurological problems. When evaluating presenting characteristics by CES-D scores below (*n* = 15) versus above (*n* = 13) the median (scores of 18–36 versus 37–48), the prevalence of higher CES-D scores was associated with male gender (*p* = 0.005), and being unemployed (*p* = 0.03), and non-significantly related to having a history of head trauma (*p* = 0.09). Medication use at study entry (data not shown) was highly variable, with 43% of subjects taking anti-anxiety medications, 32% taking anti-depressants, and 25% taking both medications. The 28 subjects were treated by six different therapists, two with doctoral level training and six with masters level training.

**Table 2:** **Demographic and presenting characteristics of study subjects**.

Characteristic	All subjects (*n* = 28)	PCL-C score at study entry*			CES-D score at study entry*		
		45 to 63 (*n* = 15)	64 to 81 (*n* = 13)	*p*-Value	18 to 36 (*n* = 15)	37 to 48 (*n* = 13)	*p*-Value
Age in years (mean ± SD)	40.5 ± 9.2	39.9 ± 8.1	41.2 ± 10.7	0.73	39.9 ± 8.2	41.2 ± 10.6	0.74
Female (%)	78.6	93.3	61.5	0.07	100.0	53.9	0.005
Caucasian race (%)^a^	92.9	93.3	92.3	1.0	93.3	92.3	1.0
Hispanic ethnicity (%)	35.7	46.7	23.1	0.25	40.0	30.8	0.71
Education (%)				0.78			0.78
High school or less	39.3	33.3	46.2		40.0	38.5	
Some college	25.0	26.7	23.1		20.0	30.8	
College degree	35.7	40.0	30.8		40.0	30.8	
Married (%)	60.7	73.3	46.2	0.25	60.0	61.5	1.0
Unemployed (%)	53.6	46.7	61.5	0.48	33.3	76.9	0.03
History of hypertension (%)	10.7	0.0	23.1	0.09	6.7	15.4	0.58
History of diabetes (%)	7.1	13.3	0.0	0.48	13.3	0.0	0.48
History of head trauma (%)	10.7	6.7	15.4	0.58	0.0	23.1	0.09
History of neurological problems (%)	17.9	6.7	30.8	0.15	20.0	15.4	1.0
Five or more traumatic memories (%)	53.6	66.7	38.5	0.25	66.7	38.5	0.25
Traumatic memories ≥11 years (%)	82.1	86.7	76.9	0.64	86.7	76.9	0.64
Guilt associated with memories (%)	89.3	86.7	92.3	1.0	86.7	92.3	1.0
Prior PTSD mental health treatment (%)	75.0	86.7	61.5	0.20	80.0	69.2	0.67
Currently on disability for PTSD or other mental health condition (%)	18.5	26.7	8.3	0.34	20.0	16.7	1.0

*Of 13 subjects with PCL-C score ≥64, 10 (76.9%) had a CES-D score ≥37; of 13 subjects with CES-D score ≥37, 10 (76.9%) had a PCL-C score ≥64; p = 0.007.

^a^*Non-Caucasian: African American (n = 1) and American Indian (n = 1)*.

### Examination of treatment efficacy

**Figure 2:**
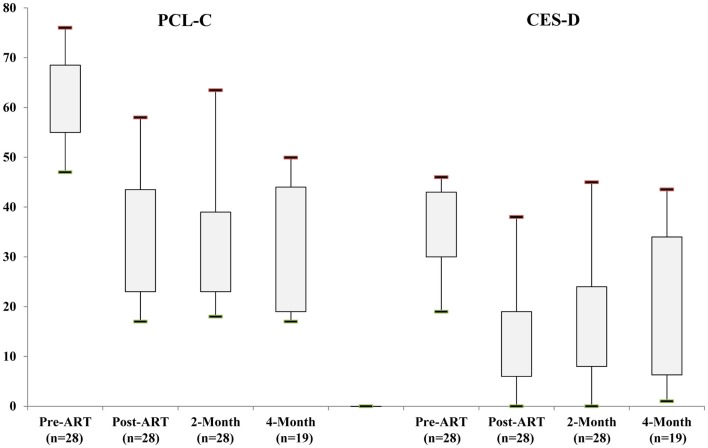
**Distribution of self-report scores on the 17-item PCL-C (left side: possible range of 17–85) and 20-item CES-D (right side: possible range of 0–60) at baseline, post-treatment, and 2- and 4-month follow-up**. The rectangles depict the interquartile range; the lower and upper ends of vertical lines depict the fifth and ninety-fifth percentiles, respectively.

For the 28 subjects who completed treatment and had follow-up data, very large, statistically significant treatment effects were observed for symptoms of PTSD and depression (Table 3; Figure 2). For the PCL-C, the pre-ART mean (±SD) was 62.5 (8.8) with mean reductions of −29.6 (12.5), −30.1 (13.1), and −31.4 (14.04) at post-ART, 2-month, and 4-month follow-up, respectively (*p* < 0.0001). Compared to pre-ART status, this corresponded to standardized effect sizes of 2.37, 2.30, and 3.01, respectively.

**Table 3:** **Mean outcome difference scores and effect sizes from pre-treatment to post-treatment, and 2- and 4-month follow-up**.

	Pre-ART (*n* = 28)	Mean difference scores (SD)			Effect sizes (95% C.I.)			*p*-Value*
		Post ART (*n* = 28)	2-month (*n* = 28)	4-month (*n* = 19)	Pre/post	Pre/2-month	Pre/4-month	
PTSD checklist (PCL-C)	62.5 (8.8)	−29.6 (12.5)	−30.1 (13.1)	−31.4 (10.4)	2.37 (1.61–3.13)	2.30 (1.57–3.03)	3.01 (1.94–4.08)	<0.0001
CES-D (depression)	35.1 (8.8)	−20.6 (11.0)	−18.1 (11.5)	−15.6 (14.4)	1.88 (1.25–2.52)	1.58 (1.04–2.12)	1.09 (0.44–1.74)	<0.0001
Brief symptom inventory	39.2 (13.7)	−25.5 (11.9)	−25.6 (13.9)	−24.7 (14.1)	2.14 (1.45–2.83)	1.85 (1.23–2.46)	1.75 (0.98–2.52)	<0.0001
STICSA (somatic)	24.0 (7.1)	−8.9 (6.8)	−7.5 (5.9)	−6.3 (5.4)	1.30 (0.76–1.85)	1.27 (0.79–1.74)	1.16 (0.62–1.71)	<0.0001
STICSA (cognitive)	28.8 (5.1)	−12.3 (5.3)	−8.9 (7.2)	−9.1 (7.2)	2.34 (1.61–3.07)	1.24 (0.72–1.76)	1.28 (0.68–1.88)	<0.0001
Pittsburgh sleep quality	11.4 (4.0)	−3.4 (3.5)	−3.2 (4.0)	−4.4 (4.2)	0.97 (0.57–1.37)	0.80 (0.40–1.20)	1.05 (0.36–1.74)	<0.0001
Trauma-related growth								
Global guilt	5.4 (2.2)	−3.3 (2.6)	−3.4 (2.5)	−2.5 (2.0)	1.27 (0.70–1.75)	1.37 (0.80–1.94)	1.27 (0.67–1.86)	<0.0001
Distress	19.8 (3.2)	−10.5 (6.1)	−10.2 (5.4)	−9.5 (5.4)	1.73 (1.11–2.35)	1.89 (1.24–2.54)	1.76 (1.00–2.51)	<0.0001
Guilt cognition	49.6 (17.4)	−21.3 (17.4)	−22.5 (18.5)	−20.9 (15.9)	1.23 (0.70–1.75)	1.22 (0.69–1.74)	1.32 (0.67–1.96)	<0.0001
Post-traumatic growth								
Relation to others	11.6 (7.0)	6.6 (6.0)	3.7 (6.0)	3.8 (6.6)	1.10 (0.65–1.56)	0.62 (0.25–0.99)	0.58 (0.07–1.10)	<0.0001
New possibilities	12.8 (6.2)	6.3 (5.6)	3.0 (6.1)	4.1 (6.7)	1.12 (0.65–1.60)	0.50 (0.14–0.86)	0.61 (0.00–1.22)	<0.0001
Personal strength	7.9 (5.4)	5.3 (7.7)	4.3 (3.8)	2.7 (4.3)	0.91 (0.46–1.36)	1.13 (0.73–1.53)	0.64 (0.23–1.05)	<0.0001
Spiritual change	5.4 (2.9)	2.1 (3.1)	0.9 (3.7)	1.8 (2.7)	0.69 (0.23–1.15)	0.24 (−0.22–0.71)	0.67 (0.18–1.16)	0.08
Appreciation-life	7.1 (4.5)	3.6 (4.7)	2.0 (4.5)	2.9 (2.9)	0.77 (0.30–1.23)	0.45 (0.03–0.88)	1.00 (0.55–1.45)	0.01
Self-compassion scale	60.5 (14.7)	20.1 (20.5)	17.1 (19.5)	13.6 (17.0)	0.98 (0.52–1.44)	0.88 (0.44–1.31)	0.80 (0.35–1.25)	<0.0001
Aggression questionnaire	81.9 (20.4)	−15.9 (14.0)	−13.9 (16.2)	−16.8 (16.9)	1.14 (0.74–1.54)	0.86 (0.49–1.23)	0.99 (0.51–1.48)	<0.0001
Alcohol use (AUDIT)	2.6 (2.7)	−0.2 (2.1)	−0.8 (2.6)	−0.7 (1.5)	0.11 (−0.20–0.43)	0.31 (−0.11–0.72)	0.47 (0.11–0.83)	0.03

*Based on repeated measures analysis of variance.

For the CES-D, the pre-ART mean was 35.1 (8.8) with mean reductions of −20.6 (11.0), −18.1 (11.5), and −15.6 (14.4) at post-ART, 2-month, and 4-month follow-up, respectively (*p* < 0.0001). This corresponded to standardized effect sizes of 1.88, 1.58, and 1.09, respectively. Thus, while there appeared to be a slight attenuation of treatment effect over time for depression (CES-D scores), effect sizes were still large and suggestive of sustained treatment response for symptoms of both PTSD and depression.

Treatment with ART showed evidence of clinically and statistically significant initial and sustained improvements across a range of psychological symptoms and measures including the BSI (effect sizes: 2.14, 1.85, 1.75), somatic anxiety (effect sizes: 1.30, 1.27, 1.16), cognitive anxiety (effect sizes: 2.34, 1.24, 1.28), and subscales of trauma-related growth. Of note, the post-treatment effect sizes for sleep quality (PSQI) and alcohol use (AUDIT) appeared to be largest at the 4-month follow-up assessment. The PSQI inquires about sleep quality over the past month, whereas the AUDIT has several questions inquiring about alcohol use over the past year (i.e., potential “delayed” treatment effect due to time period for which symptoms were reported).

**Figure 3:**
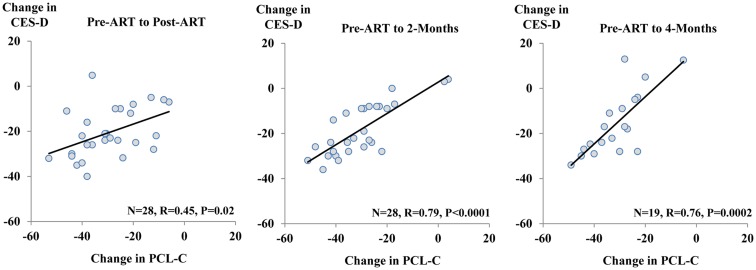
**Plots of change in PCL-C scores (*x*-axis) and change in CES-D scores (*y*-axis)**. The plots are presented as changes from pre-ART to post-ART (left side); pre-ART to 2-month follow-up (middle); and pre-ART to 4-month follow-up (right side). Pearson correlation coefficients are listed.

The relationship between change in scores on the PCL-C and change in scores on the CES-D was modest when considering pre-ART to initial post-ART assessment (Figure 3; *r* = 0.45, *p* = 0.02). However, much stronger relationships were evident for changes in PTSD and depression scores from pre-ART to 2-month follow-up (*r* = 0.79, *p* < 0.0001) and pre-ART to 4-month follow-up (*r* = 0.76, *p* = 0.0002). Of note, completion of questions on the PCL-C is for the past month compared to the past week for questions on the CES-D. In examining change in PCL-C symptom subscale scores pre-ART to 2-month follow-up and corresponding changes in CES-D scores, modest to strong relationships were consistently observed: intrusion/re-experiencing (*r* = 0.65, *p* = 0.0002); avoidance (*r* = 0.47, *p* = 0.01); numbing (*r* = 0.60, *p* = 0.0008); hyperarousal (*r* = 0.70, *p* < 0.0001). These results are consistent with the postulate that depression and PTSD may actually represent a singular construct (O’Donnell et al., 2004; Gros et al., 2010; Elhai et al., 2011).

### Adverse events

One participant reported feeling depressed the day after an ART session which subsequently resolved. This participant completed treatment and follow-up. Three additional non-study (treatment) related events were reported: (i) light headedness with poor balance and memory; (ii) headache at the time of the 2-month follow-up due to having five teeth pulled; and (iii) delayed ability to complete 2-month follow-up questionnaires due to a car accident.

### Sensitivity analysis

Of the 35 participants enrolled, 7 (20%) did not complete treatment (*n* = 4) or completed treatment yet without follow-up (*n* = 3), thereby representing a potential source of bias. For the PCL-C, a reduction of ≥10 points from baseline assessment has been defined to represent “reliable” and “clinically meaningful” change (Monson et al., 2008). For the 28 participants with complete data, 26 (92.9%) achieved this metric; assuming the worst case scenario whereby the remaining 7 participants failed to respond, this would result in a minimum response rate of 74.3% for reliable and clinically meaningful change in symptoms on PTSD. For the CES-D, a reduction of ≥5 points from the baseline assessment has been defined to represent reliable and clinically meaningful change (Jacobson and Truax, 1992). For participants with complete data, 89.3% achieved this metric; assuming the worst case scenario whereby the remaining seven participants failed to respond, this would result in a minimum response rate of 71.4% for meaningful change in symptoms of depression.

## Discussion

In this uncontrolled prospective cohort study of adults with comorbid symptoms of PTSD and depression, treatment with ART was associated with large, concomitant reductions in symptom scores. These effects were observed immediately post-treatment (effect sizes of 2.37 and 1.88) and at 2-month (effect sizes of 2.30 and 1.58), and 4-month follow-up (effect sizes of 3.01 and 1.09). Clinically, results were achieved with a mean of 3.7 ART sessions. Among current first line evidence-based treatments for PTSD (irrespective of comorbid depression), CPT entails 12 treatment sessions (Resick and Schnicke, 1996), PE therapy typically requires 8–15 treatment sessions (Foa and Rothbaum, 1998), and eye movement and desensitization (Chemtob et al., 2000; Shapiro, 2001) is an 8-phase program that entails 5–15 treatment sessions. To address overall length of treatment, shorter psychotherapies have been investigated, particularly during the acute phase of PTSD, and with limited empirical evidence of benefit (Sijbrandij et al., 2007; Rothbaum et al., 2008). Given the brevity of ART, application to comorbid symptoms of PTSD and depression, and large effects observed, these data provide a rationale for mechanistic studies and formal comparative effectiveness studies of ART versus established evidence-based psychotherapies.

### Possible treatment effect mechanisms

Whereas ART (developed in 2008) is an emerging new therapy with a limited empirical evidence base to date (Kip et al., 2012), multiple explanations may explain, at least in part, the apparent clinically meaningful treatment effects of ART on reducing comorbid symptoms of PTSD and depression, as well as the close concordance between post-treatment changes in these symptoms. At the broadest level, these explanations can be classified distinctly (i.e., not by relative order of importance) as diagnosis-based, temporal-sequencing, and therapeutic (mechanistic)-based.

First, among trauma-exposed individuals, it has been shown empirically that PTSD and MDD symptom items may represent an underlying dimension, rather than two separate dimensions (Gros et al., 2010). Gros et al. (2012) recently reported that patients classified as having either PTSD or MDD (but not both) on the basis of the Clinician Administered PTSD Scale (CAPS) (Blake et al., 1995) and Mini International Neuropsychiatric Interview (MINI) (Sheehan et al., 1998) had comparable PTSD symptom levels, as derived from the PCL-C and Trauma Assessment for Adults Questionnaire. Thus, it is not unexpected that changes in symptoms of both PTSD and depression after treatment (i.e., with ART) would be highly correlated. These data also support the notion of broader use of transdiagnostic treatments that emphasize the commonality of emotional disorders and highly overlapping symptoms (Ellard et al., 2010).

Second, PTSD may precede depression (Breslau et al., 1997, 2000), and vice versa, depression may precede PTSD (Shalev et al., 1998; Erickson et al., 2001). King et al. (2009) prospectively studied adult emergency room admittees who experienced a traumatic event and provided evidence for both directions. However, when PTSD was antecedent to depression, the strongest relationship occurred for PTSD symptom clusters of intrusion and hyperarousal, and little to no predictive value of the PTSD symptom clusters of avoidance and numbing. By design, our study recruited and enrolled participants on the basis of presenting PTSD symptomatology, and ART was initially conceived and developed for the treatment of psychological trauma. In spite of this, we cannot ascertain the extent to which PTSD preceded symptoms of depression in our study sample. Nonetheless, we observed strong pre/post-treatment relationships (improvements) between all PTSD symptom clusters and depression. Therefore, at least with respect to treatment with ART, reductions in depressive symptoms appear to occur in close concordance with reductions in all symptoms of PTSD.

Third, in addition to use of the VIR technique in ART for imagery rescripting, it is possible that lateral left-right eye movements may foster inter-hemispheric communication in a “problem-solving” manner similar to that used during rapid eye movement (REM) sleep. A hallmark of PTSD is disrupted sleep with intrusive, terrifying dreams and defective processing of emotionally laden memories (Foa and Kozak, 1986). Similarly, sleep structure in depression, in comparison to healthy individuals, is characterized by reduced slow-wave sleep (SWS), shortened REM sleep latency, and redistribution of REM sleep throughout the night (Coble et al., 1981; Kupfer et al., 1984). Moreover, both the PCL-C and CES-D have specific sleep-impairment items. Theoretically, if the sets of eye movements used with ART “simulate” some aspects of REM sleep, this in turn may be expected to result in more selective and efficient processing of emotional memories (Wagner et al., 2001) with therapeutic benefit for both PTSD and depression.

A hypothesis alternative yet similar to imagery rescripting to address how ART appears to produce salutary effects on treatment outcome is that ART interacts with a process known as “memory reconsolidation.” Extensive research indicates that a fear-provoking memory is first stored, i.e., consolidated, and then after it is retrieved at a later time it must be actively stored once again, or reconsolidated (Nader et al., 2000; Nader and Hardt, 2009). Preclinical (Nader et al., 2000; Duvarci et al., 2005; Pitman, 2011; Finnie and Nader, 2012) and clinical (Brunet et al., 2008, 2011; Schwabe et al., 2012) studies have shown that during the reconsolidation process, a memory is vulnerable to modification by amnestic or anxiolytic agents administered in conjunction with traumatic memory reactivation, i.e., during exposure therapy. Therefore, pharmacological blockade of reconsolidation has become a novel strategy in the treatment of PTSD (Debiec and LeDoux, 2006; de Quervain and Margraf, 2008; Donovan, 2010; Pitman, 2011). The therapeutic benefit of ART for both PTSD and depression may be related to findings in research on reconsolidation. In theory, the interaction of imaginal exposure that occurs in conjunction with the critical elements of ART, i.e., eye movements, reduced physiological and affective responses during imaginal exposure, and imagery rescripting, generates a reconsolidated hybrid memory, which includes non-trauma (ART-specific) components. The hybrid memory, therefore, upon subsequent retrieval, may be less traumatic to the patient.

### Strengths and limitations

There are several strengths to our study. All therapists were formally certified in ART using a standard protocol, recruited from the community, and possessed varying educational backgrounds at the masters and doctoral level. This provides some degree of generalizability across therapists, as well as standardization of the training and delivery of ART. However, despite completing a standardized 42-item ART fidelity checklist after each treatment session, there was no independent verification of delivery of ART by the six individual clinicians. Thus, this study cannot confirm that all clinicians delivered ART in full compliance with the treatment protocol. Second, the self-report outcome measures utilized are reliable and valid instruments. Third, neither the founder of ART (Laney Rosenzweig) nor lead trainer (Amy Shuman) performed any cases or participated in data collection or analysis.

Despite these strengths, there are limitations. First and foremost, there was no control group to compare the results achieved with ART versus therapist attention alone. This uncontrolled cohort study design is typical for an early stage evolving therapy such as ART, but can provide suggestion of effectiveness. Second, assessment of treatment efficacy of ART for comorbid PTSD and depression is based on changes in symptomatology. Thus, we cannot directly infer from our data that use of ART results in change in DSM-derived diagnoses, including PTSD and depression. Having said this, the multiple symptom criteria used in this analysis (see Materials and Methods) to define clinically significant PTSD and depression provide some assurance of true diagnoses in the majority of participants. Third, subjects presented with high levels of symptomatology beyond PTSD and depression (e.g., somatization and OCD) which limits generalizability of study results to confined presentations of PTSD and depression, yet, is also characteristic of multiple dimension comorbidity frequently seen in clinical practice. Finally, the sample size of 28 subjects is modest, predominantly female, and 9 did not complete the 4-month follow-up assessment. These characteristics limit broad generalizability.

## Conclusion

From this uncontrolled prospective cohort study, ART appears to be a brief, safe, and effective treatment for adults with clinically significant comorbid symptoms of PTSD and depression. Future mechanistic and controlled studies with ART are warranted, particularly given its much shorter treatment protocol in comparison to current evidence-based therapies.

## Conflict of Interest Statement

The authors declare that the research was conducted in the absence of any commercial or financial relationships that could be construed as a potential conflict of interest.

## Author Note

The Principal Investigator and primary statistician of the study, Kevin E. Kip, Ph.D., takes responsibility for the integrity of the data and the accuracy of the data analysis. Ms. Laney Rosenzweig is the developer of ART and has a commercial interest in its dissemination. She did not perform any cases or participate in data collection or analysis.
